# Elite and Liberal Democracy: A New *Equilibrium*?

**DOI:** 10.1007/s11245-021-09762-1

**Published:** 2021-10-11

**Authors:** Antonio Campati

**Affiliations:** Dipartimento di Scienze Politiche, Facoltà di Scienze Politiche e Sociali, Università Cattolica del Sacro Cuore, Largo Gemelli, 1, 20123 Milano, MI Italy

**Keywords:** Elite, Liberal democracy, Disintermediation, Democratic distance

## Abstract

In contemporary democracies, the balance between the *minority principle* and *democratic principles*, one of the components underlying the relationship between liberalism and democracy, is being broken. This paper offers a reflection on this theme – crucial for the future of representative government – in relation to the importance of the theory of elites. The article is divided into three parts: the first part briefly traces the main phases of the theory of elites from the late nineteenth century to the present, indicating, for each, the salient features; the second part focuses on the elements characterizing the alliance between the minority principle and democratic principles, which forms the basis of liberal representative democracy, with specific consideration paid to the geometric architecture of democracy, comprising a horizontal dimension and a vertical dimension; finally, the third part argues the need for strengthening the *logic of distance* to consolidate the connection between the theory of elites and liberal representative democracy.

## Introduction

In recent years, more and more scholars have pointed out that liberal democracy is *under pressure* (Galston 2020). There are those who argue that there is an *anti-liberal revolt* (Krastev and Holmes 2020) capable of penetrating most Western countries and thus radically transforming their foundations. Others emphasize the split between the people and democracy, warning against the spread of forms of *illiberalism*, already significantly present in countries considered until recently as exemplary democracies (Mounk 2018). Moreover, there are some who, even more drastically, argue that after the spread of democracy and its liberal ideals, especially following the fall of the Berlin Wall in 1989, there has been a *counter-revolution* (Zielonka 2018), which would testify to a real failure of liberalism, both as a doctrine and as a model for the definition of an international order (Deneen 2018; Ikenberry 2018; Toplisek 2019).

Despite the different meanings, there is no doubt that liberal democracy is effectively *under pressure*. In fact, the causes of such a radical rejection of what was considered the political model of reference, capable of guaranteeing citizens’ freedom, equality and well-being, are many and varied. Within this framework, this article focuses on one of the main causes of this *democratic malaise*, namely the breaking of the balance between the *minority principle* and *democratic principles*. This balance has legitimized – at least from the second half of the twentieth century onwards – the idea that in liberal democracy, it is a minority (elites) that govern over the majority. Today, there are several reasons that lead citizens to consider this idea outdated, including the poor quality of the political class that provokes its rejection and the emergence of forms of disintermediation, including the opportunities for direct dialogue with political leaders guaranteed by social media, which makes the presence of mediators useless. One of the most evident confirmations of all this is the success of populist leaders who base their political proposals precisely on the clear separation between corrupt elites and *pure* people, proposing themselves as the only ones able to fight the former and defend and represent the latter.^1^

However, a study of the role of elites cannot be limited only to the analysis of populism, the use of social media or the denunciation of the poor quality of elected officials. These (and other) elements are certainly useful for an understanding of the transformations of contemporary democracies, but the *rupture* of the *balance* between the minority principle and democratic principles deserves specific attention because it is the basis of all the other manifestations of disaffection towards elites. This paper aims to demonstrate the reasons for this claim and is structured in three parts: the first briefly traces the main phases of the theory of elites from the late nineteenth century to the present day, indicating, for each, the salient features; the second focuses on the elements characterizing the *alliance* between the minority principle and democratic principles, which forms the basis of liberal representative democracy, with specific attention paid to the *geometric architecture of democracy*, consisting of a horizontal dimension and a vertical dimension; the third formulates the hypothesis of strengthening the *logic of distance* to consolidate the connection between elite theory and liberal representative democracy.

## The Phases of Elite Theory

As is well known, elite theory aims to explain in a scientific manner one of the indisputable regularities of human history: “the fact that, in every age and in every society, a numerically small segment of people tends to concentrate in their own hands a high quantity of resources and to impose itself on almost all of the population who lack them” (Sola 2000, 7). Clearly, an unequal distribution of the possession and control of resources results in an equally unequal distribution of power. The classical elaboration – especially with respect to the political sphere – by Gaetano Mosca expresses this idea. Mosca underlines how in all social organizations, there are two classes of people: the rulers and the governed. The former are less numerous and capable of monopolizing power, while the latter are more numerous and directed by the first in an arbitrary and violent way (Mosca 1982, 608).^2^

If this is a regularity in all societies, it is thus also the case in democracies, where elite theory can be subject to alteration. The theory can be subjected to *ideological use*, above all to contest the myth of self-government and the institution of universal suffrage or, by contrast, to enhance competition, the selection of politicians and control of their work (Sola 2000, 11). Over the past few decades, such an ideological use of elite theory has somehow become even more accentuated, especially with the aim of *weakening* the actions of elites in order to enhance the proper functioning of liberal democracy.^3^ To better understand this trend, it is necessary to broadly retrace the stages in the history of the relationship between elite theory and democracy, which can be categorized into at least three major phases (Tuccari 2017, 129). Between the nineteenth and twentieth centuries, the first theories of elites stood *against* democracy, as they were born in reaction to the advent of mass politics, political professionalism, organized political parties and, more generally, the crisis of the traditional arrangements of representative systems. Later, in the period between the two wars, with the birth of fascism, Nazism and communism, theories of elites “could and should have become a kind of garrison of liberal democracies and plural society” (Tuccari 2017, 129); hence, they were theories *for* democracy. Finally, today, it is increasingly difficult to combine elitism and democracy, and it is therefore probable that we must think of a theory of elites that goes *beyond* democracy.^4^

During the first phase of elite theory, democracy was seen above all in problematic terms, that is, as a dangerous consequence of the gradual widening of the participation of the masses in political life.^5^ However, some authors, while retaining a great deal of scepticism towards the forms of enlargement of suffrage, try to imagine the hypothesis of a conciliation between the minority principle and democratic principles. In other words, there is an effort to find a theoretical framework that allows the laying of the foundations for a compromise between the inevitable presence of elites in power and forms of democratic participation. To achieve such an ambitious goal, it is necessary to emphasize an essential starting point highlighted in 1865 by a little-known author, Ruggiero Bonghi, who noted the clear distinction between allowing everyone to *aspire* to political leadership and the possibility of *making access* common (Bonghi 1865). In this sense, the outline of the theory of political elites is modelled, with a minority of rulers holding political power and, above all, prevailing over the majority by being organized. The features of the theory of the political class began to emerge, which Mosca, two decades later, systematically established in *The Ruling Class* (*Elementi di scienza politica*).^6^ Thanks to his studies, as well as those of other *classical* elitists such as Pareto and Michels (Drochon 2020), the foundations of a very broad, albeit partially disorganized, body of doctrine were laid.

In the following years, by developing and precisely criticizing these elaborations, several authors inaugurated the second phase of the theory of elites, the one in which the latter are considered a defence for democracy. Thus, there is a real reconciliation between elitism and democracy (Stoppino 2000, 259) through a series of proposals that are not always convergent; indeed, they are at times very different from one another. One of the best known is that of Joseph A. Schumpeter proposed in *Capitalism, Socialism and Democracy* (1942), which derives from the elitist tradition. With a special emphasis on the theme of elites competing for power, this represents one of the most significant expressions of *democratic elitism*. As is well known, this expression is rather equivocal, above all because it is a variant of the elite theory formulated by Mosca, Pareto and Michels. Each of these three authors belongs to different cultural traditions: the first two can be considered *liberal* but certainly not *democratic*, while the third starts his analysis from *ultra-democratic* assumptions, with very few *liberal* assumptions (Tuccari 2017, 116). This is relevant because the *democratic elitism* that develops in the second phase of elite theory is characterized by a diversity of views on a crucial aspect, namely what is meant by democracy. Moreover, the expression “democratic elitism defines in a generic way a conception of politics expressed in variable forms and content by scholars of different intellectual backgrounds, who all profess to be democrats but each refer to a different idea of the meaning of democracy” (Bovero 1975, 22).^7^

The nature of the debate that has developed around *democratic elitism* is not unrelated to the subsequent discredit that has struck elites. Indeed, the most ferocious critics of the neo-elitist position accuse elites of proposing a reversal of the people–elite dynamic, repudiating an ethical ideal of democracy and thus reducing it to a political method for the choice of leader.^8^ All these trends gradually accentuated until they exploded for approximately three decades in a tireless debate on the *crisis* of democracy, which sees the presence of elites as a weak point, after having played an essential role in the construction of contemporary democratic theories (as noted by Tuccari 2016, 4).

Despite all these changes, however, it is difficult to found the beginning of a “new phase” of elite theory on the hypothesis that elites must disappear. It should not be forgotten that the economic elites continue to play an important role (Di Leo 2012) and their influence in large areas of society (Wolin 2008) is confirmed by many empirical studies; in the same way, through elections – one of the requisite elements for democracy – citizens select representative elites; moreover, the epistocratic elites continue to influence the politics of states (think of their role during the Covid-19 crisis); and even the populist leaders – despite the rhetorical hatred they pour out towards the elites – are in all respects part of it. The “new phase” of elite theory must therefore not focus on the hypothesis of their end (nor on the end of democracy). The *new equilibrium* between elite and liberal democracy must be analysed starting from the mismatch of the *previous equilibrium*, that between the vertical dimension and the horizontal dimension of democracy.

## The *Previous Equilibrium*: A Geometric Theory of Democracy

The balance between the minority principle and democratic principles represents the heart of liberal representative democracy. To understand the importance of this link, it is appropriate to start from one of the various models proposed in the second phase of elite theory. Certainly, among the most important is that recommended by Giovanni Sartori, considered one of the leading exponents of neo-elitism (Valbruzzi 2017, 64). Here, we are interested in recalling just one aspect of the considerable work of this Italian scholar (Pasquino 2019, 97–192; Passigli 2015): his *geometric* conception of democracy, which develops in a *vertical dimension* and a *horizontal dimension* (Valbruzzi 2017).^9^ According to Sartori, vertical democracy is “democracy as a system of government”, whereas the vision of politics “horizontally” is the anarchic one, that is, that of non-command (Sartori 2011, 91). The development of these two dimensions has a genesis that began in ancient Greece and found a turning point at the turn of the nineteenth and twentieth centuries, just when the first phase of elite theory had been accomplished. It is in this phase that, with the entry of the masses into political life, the horizontal dimension – the dimension of popular participation, especially through the expansion of suffrage – manages to counterbalance the vertical dimension of power, that is, the sphere of command (Sartori 1995, 273).

Based on this outline, reflection on the importance of elites becomes crucial, especially since the use of electoral procedures – that is, the way in which the horizontal dimension is strengthened – does not escape *altimetric discourse*, as such discourse is indispensable for recruiting the personnel who will go on to work in political fora. Therefore, as Sartori clarifies, the diffusion of politics does not take place “only at the basic level, at the level of the demos”, but also “at the level of the elites”; in fact, democracies “are structured as competitive” polyarchies “with a wide pluralistic dissemination” (Sartori 1995, 274).

This precise conception of democracy is the basis for consolidating the reconciliation between elitism and democracy, which, as mentioned, is the main feature of the second phase of elite theory. This *reconciliation* was possible because there was an important *union* between the *aristocratic nature of liberal individualism* and *social democracy*, that is, between liberalism and democracy, where the former focuses on the *individual* and the latter on *society*. According to this idea, “liberalism has a vertical impetus […] while democracy is horizontal diffusion” (Sartori 1995, 145–146).^10^ Therefore, what we mean by the term *liberal democracy* is the result of the union between “individualizing and uplifting liberalism” and “inclusive and levelling democracy” (Valbruzzi 2017, 62).^11^ In this sense, Sartori’s attention to *democratic verticality* is becoming increasingly accentuated, probably for two reasons: first, to deepen the analytical dimension with respect to the link between the verticality of democracy and the system of representation; second, for value and regulatory issues, reflecting the need to emphasize the danger that occurs when the *elitistic element* is neglected and with the aim of leaving more space for the participatory dimension. These two aspects should be considered because they help us understand the current *crisis* in which liberal democracy finds itself. As mentioned, for Sartori, elections represent the point of intersection between public opinion (which develops horizontally) and the sphere of authority (which, instead, is structured vertically). Therefore, liberal democracy is based on a representation theory that is not just competitive but a *competitive-feedback theory*, that is, a competitive struggle that produces feedback (Valbruzzi 2017, 63), which thus presents a strong distinction between the figure of the representative and that of the represented.^12^ From a regulatory point of view, however, Sartori complains of a lack of a drive to discover an ideal for vertical democracy: this “was built, or in any case left, *without valuable support*. The result is not only an altimetry *without ideals*, neither supported nor fed by its own values, but also almost defenceless when attacked by horizontal values” (Sartori 2011, 114).

In this sense, Sartori points the finger at the silence of the *verticalists*, whereas, by contrast, the supporters of horizontal forms of democracy have been better able to affirm their positions.^13^ These have even favoured the spread of *counter-ideals*, that is, “values which have as their aim that of compressing all democratic verticality to the maximum” (Valbruzzi 2017, 66). Moreover, such a rejection of the vertical dimension of democracy is evident both in proposals that focus on the *direct* participation of citizens in political life (e.g. through Informations and Communication Technologies) and in the persistent negative connotations of the very term elite. More specifically, the two main theories that attack liberal democracy head-on are epistemic approaches to democracy and the populist challenge: in the first case, the goal is “to untie the sphere of command from the horizontal sphere of public opinion”, while in the second case, there is a tendency to “lower” the vertical dimension to the level of the horizontal one (Valbruzzi 2017, 67–68).^14^ These are two temptations that constantly threaten liberal representative democracy, as it consolidated in the Western world, especially during the second half of the twentieth century. Today, liberal democracy is faced with an even more demanding challenge because the vertical dimension has weakened more than when Sartori expressed his concerns. For example, due to the use of IT platforms, which, to allow new forms of non-mediated political participation have weakened political representation understood as a mediated relationship. In the same way, the union between individualizing liberalism and inclusive democracy is in serious difficulty and the various forms of illiberalism that are developing in European and extra-European countries offer proof of this. In other words, the balance between the horizontal and vertical dimensions of democracy is now compromised, so the whole architecture that supports liberal representative democracy risks collapsing.^15^

## A New Phase: Reiterating the *Logic of Distance*

One of the main causes of this hypothetical collapse is due to the claim to found an “integral democracy”, that is, a democracy that calls into question its constitutive *equilibrium*. Norberto Bobbio highlights how the theory of elites presents itself with a *realistic face* and an *ideological face*: in the first case, it maintains the thesis according to which power always belongs to a minority and the only difference between one regime and another lies in the presence or absence of competing minorities; in the second case, its main *historical function* is that of “denouncing from time to time the ever-reviving illusions of an integral democracy”, as it was born as a reaction to the feared advent of mass society (Bobbio 2004, 347).^16^

Despite the difficulties that arise from such a polarity,^17^ trends in contemporary societies lead us to argue that elite theory can make a further contribution to democracy studies. Indeed, in accordance with Bobbio’s words, it is useful once again to *denounce* the illusions of a possible *integral* democracy, which today takes the form of a hypothetical *immediate democracy*, that is, a democracy without mediation, with the illusion of drastically reducing representation.^18^ Therefore, it allows us to reaffirm the evidence that power is concentrated in the hands of a few, despite the pressure from forms of participation from below that tend to weaken the vertical dimension of democracy.

To discover the theoretical and empirical limits of the *illusion* that comes from immediacy, it is necessary to revive the *logic of distance*, which is closely connected to the representative principle of distinction. Nadia Urbinati notes that “if in the past distance was an obstacle to participation, today it becomes a vehicle to facilitate reflection and autonomy of judgment” (Urbinati 2013, 176). Indeed, just when disintermediation processes^19^ have become more evident, to maintain the balance of representative democracy, it should be remembered that it is precisely *distance* that functionally characterizes the relationship between the representatives and the represented (Rosanvallon 2015, 289). This relationship is by no means peaceful but is constantly looking for a balance structurally characterized by two contrasting logics, which must precisely find a point of synthesis: a *logic of proximity* that obliges the representatives to keep in touch and listen to citizens and a *logic of distancing* that invites them, on the contrary, to keep away from them (Innerarity 2020, 162). In short, the distance that separates those who are inside the “decision-making palace” and those who are outside it is “the space of play through which contemporary democracy is built”: it is the place where the conflicting relationship between “the many” and “the few” takes place (Urbinati 2020b, 90).^20^ This balance between *proximity* and *distancing* is therefore fundamental for thinking about an elite theory in a context such as that of a contemporary democracy, where, as we have just recalled, “the elimitation of mediation” is an imperative request, although deeply ambiguous (Innerarity 2020, 161–162).^21^ This tends to make the logic of proximity prevail in cancelling not only the distance but also the differences between representatives and represented.

Indeed, it must be remembered that representative government was established in full awareness that elected representatives would be separate from citizens and socially different from those who elected them, thus affirming what Bernard Manin has referred to as the “principle of distinction” (2010, 105). The ambiguous nature of the presence of elites in a democracy certainly resides in this principle (Leboyer 2016, 2012; Rosenberg 2020),^22^ above all because it strikes a raw nerve in democracy due to the unequal distribution of power. Is it possible that in a democratic regime only the *few* make binding decisions for *all*? This question emerges in an increasingly nagging manner, especially when the concentration of power in the hands of a small elite is considered simply the result of an oligarchic tendency and not the result of the application of the *principle of distinction*. The presence of elites in a representative democracy – and thereby the unequal distribution of power – must be studied in relation to the *logic of distance*. Daniel Innerarity has stressed that, in this case, a balance must be maintained between *mediation* and *disintermediation* to ensure equality and citizen participation in public life: “mediation provides equality; disintermediation provides the voice. Equality and voice are both essential democratic values; each provides a correction to the other. Disintermediation corrects for exclusion; mediation corrects for inequality” (Innerarity 2019, 522). Representative democracy is therefore a political model based mainly on these two mediations, which find their most evident concretization in the distinction between representative elites and the people represented. This distinction does not simply delimit a *space* but is the cornerstone of a *logic* that relates the *few* and the *many* through a series of stable and structured mediations thanks to the presence and actions of intermediate bodies, thereby providing full legitimacy to elite theory. According to the theorists of liberal democracy, the *logic of distance* is a solid glue for the union between individualizing liberalism and inclusive democratic principles (Sartori 1995, 145–146), which thus manages to calibrate the *aristocratic principle* and *democratic principles*.^23^

## The Necessary Balances of Democracy

At this point, the concerns related to the precarious relationship between democracy and liberalism and, consequently, to the *uneasy alliance* between democracy and representation (Pitkin 2004) should be more understandable, as is testified by the renewed interest in the relationship between elites and democracy (Gaxie 2017; Nodia 2020; Körösényi 2018; Vogel et al. 2019). It must be acknowledged that the breaking of the balance between the *minority principle* and *democratic principles* can lead to forms of political regimes that are very different from liberal democracy. On the other hand, although the “internet revolution” did not bring all the improvements it had promised (Runciman 2019, 133), it has nevertheless legitimized new forms of democratic participation, which are in competition, so to speak, with the electoral moment, that is, the point of conjunction between the vertical dimension and the horizontal dimension. Obviously, the strengthening of the first dimension^24^ must not lead to the weakening of the second; therefore, we must not at all envisage the downsizing of new forms of political participation, neither those more directly linked to the potential of ICT nor those with a longer tradition (Campos and André 2014).

Rediscovering the nature and logic of distance – characterized by the *balance* between *proximity* and *distancing* and between *mediation* and *disintermediation* – is thus the way to go to re-establish the balance of representative government. Elite theory helps us once again to underline the complexity of democracy, especially when it warns us of *illusions* such as that representation can be overcome by establishing an immediate relationship between a leader and their followers. As we have seen, such a wish contrasts with the nature of representative government, which involves the determination, through elections, of a minority that governs and a majority that is governed. This does not mean that the forms of immediacy are not present within a democracy, but these must be in balance with the forms of mediation. In other words, it is necessary to take seriously the fact that citizens of contemporary democracies are no longer satisfied with slipping a ballot paper into a box and prefer increasingly interactive forms of representation.^25^ At the same time, these requests are compensated for by the *logic of distance*, which characterizes the liberal democratic system through a complex series of institutional (parliaments), political (political parties) and social (associations and intermediate bodies) mediations. To reinforce this framework, it is necessary to revive the theory of elites since only by rediscovering their function will it be possible to preserve liberal democracy from the threats that haunt it.

## Footnotes

1 Among the books that highlight the contrast between elites and the people in relation to populism, see Hayward (1996), Meny (2019) and Urbinati (2020a).

2 On the theory of elites, see AAVV (1961), Albertoni and Bagnoli (2001), Bobbio (2004), Bobbio (2005), Bovero (1975), Busino (1992), Higley (2018), Stoppino (2000) and Tuccari (2002).

3 Ornaghi underlines that the concept of elites was designed to be rigorously *scientific*, but it turned immediately into a polemical political category that was thus easily and ideologically manipulable (Ornaghi 2013, 331). On the scientific and ideological dimensions of the theory of political elites, see also Ripepe (1971).

4 According to Tuccari (2017), the author who was the first to clearly formulate the idea of an *elitist school* that is at the same time realistic, democratic, and liberal was James Burnham, author of *The Managerial Revolution* (1941) and *The Machiavellians: Defenders of Freedom* (1943). On Burnham, see Borgognone (2000).

5 Placing the birth of elite theory between the end of the nineteenth century and the beginning of the twentieth century is now widely accepted by political theory. However, we must not forget that there have been significant *precursors* of this theory, such as Saint-Simon, Comte, Tocqueville and Taine (Sola 2000), as well as the contributions of other figures such as Calhoun (Salvadori 1996) and Bagehot (Easton 1949).

6 It is important to remember that Mosca establishes the origins of the doctrine of the political elite well before his work, as he explains by recalling, for example, Machiavelli, Saint-Simon and Comte (Mosca 1982, 929–940).

7 The work that puts forward a very strong criticism of democratic elitism is that of Barach, who writes explicitly that “as a political theory, it is fundamentally wrong” and that “on the normative level it fails to penetrate the essential political needs of the man of the twentieth century” (1967). In his introduction to the Italian edition of Barach’s book, Stoppino (1974, XIV) writes as follows: “From whatever perspective you look at it, the expression ‘democratic elitism’ or ‘elitist theory of democracy’ does not appear insulting or incorrect. It is another way of saying ‘polyarchic theory’ or ‘pluralistic theory of democracy’; since in a polyarchy political power is exercised by an alternation of different mobile and open elites, and the influence that the people as an electorate operates over the government depends on the pluralism of the elites and the competition that arises between them.” A critical reading of Barach’s reflections is Palano (2015). On the relationship between elites and democratic theory, see Perry (1969). For a more recent analysis of democratic elitism, see Best and Higleu (2010), but note also Bealey (1996).

8 A synthesis of the specifics of democratic elitism according to Barach (1967) is found in Stoppino (1974, XV). See also Palano (2015), Piano (2019) and Portinaro (2018).

9 On the tension between the vertical and horizontal dimensions of politics, in addition to Sartori (1979), see Bartolini (2018).

10 Furthermore, Sartori (1995, 146) underlines that liberalism and democracy, after a period of long convergence, have begun to divide again, taking “two divergent paths”. He specifies that “cracks arise when the components of liberal democracy become unbalanced, that is, when more democracy is demanded at the expense of less liberalism”.

11 See also Higley and Burton (2006).

12 On the debate on the transformations of political representation, see Castiglione and Pollak (2019), Damele (2017) and Diehl (2019).

13 Floridia clarifies that Sartori “appears strongly controversial especially towards those versions of participatory democracy which, in reality, appealed to some direct and immediate connection between participation and decisions” (Floridia 2017, 85).

14 On this point, see the final remarks of Campati (2016).

15 Manin (2010, 259) writes as follows: “the currently prevailing impression of crisis reflects the disappointment of previous expectations about the direction of history. In that its base has expanded enormously, representative government has, since its establishment, undoubtedly become more democratic. That trend has not been reversed; history has confirmed what had been believed. However, the democratization of representation, the narrowing of the gap between representatives and represented, and the growing influence of the wishes of the governed on the decisions of those in government have turned out to be less durable than expected. While one can certainly say that democracy has broadened, one cannot say with the same certainty that it has deepened.”.

16 Bobbio (2004, 347) specifies as follows: “if in its ideological face it may have contributed to hindering the advance of a democratic transformation of society (even in the sense in which democracy and the existence of a minority political class are not incompatible), in its face realistic it has contributed, and continues to contribute, to discovering and exposing the fiction of ‘manipulated democracy.’”.

17 The double declination through which the theory of elites can be understood remains a fixed point for studies in this area, but, at the same time, it also represents its limit because it highlights the difficulty of identifying the clear systematic nature of the elements that characterize it, as already found in pioneering studies (Bottomore 1967, 77; Wodak 2017).

18 On the origin of the expression *immediate democracy*, which can be traced back to Condorcet, see Rosanvallon (2015, 173) and, on the most recent developments, Rosanvallon (2020, 152–155).

19 See Chadwick (2007) and also Cuono (2005) for the distinction between *horizontal disintermediation* and *vertical disintermediation*.

20 Urbinati (2020b) adds, “when in the democratic context we use words like ‘the few’ and ‘the many’, we must avoid thinking that we are talking about two homogeneous and distinct groups of citizens, as if they were two peoples” (94).

21 According to Innerarity (2020, 160–161), “accessibility of information” gives rise to “the dream that opinion polls can make political wishes perfectly apparent, making it possible to govern based solely on polling numbers. But a ban on mediation could also produce the nightmare of a public space with no limits, procedures or representation”.

22 According to Salvadori (2011, 67), liberal democracy appears, on the one hand, as an irresistible force but, on the other, as a form of manipulation of political participation by narrow oligarchies.

23 Pizzorno (1972, 43) points out that the *equilibrium variables* of the theory of political elites vary precisely as these two principles vary: when the aristocratic principle prevails, the political class is closed; when democratic principles prevail, the political class is much more open.

24 For example, Azzolini (2017, 144) argues that “the theory of elites is not only compatible with democratic theory, but is also compatible with a progressive democratic theory, that is, attentive to the civil and social dimensions”.

25 As Rosanvallon (2015, 274) writes, “proximity is no longer considered as a variable of position, which can be adequately explained through a statute (that of the elected), but as a quality of interaction”.
